# Efficient uranium immobilization on red clay with phosphates

**DOI:** 10.1007/s10311-013-0442-2

**Published:** 2013-10-30

**Authors:** Ewelina Grabias, Agnieszka Gładysz-Płaska, Anna Książek, Marek Majdan

**Affiliations:** Faculty of Chemistry, Maria Curie-Skłodowska University, PL MC Skłodowskiej 2, 20-031 Lublin, Poland

**Keywords:** Uranium, Phosphates, Red clay, Arsenazo III, Phosphomolybdic acid, Sorption percentage, Isotherms, Distribution constant, Surface complexes, X-ray photoelectron spectroscopy

## Abstract

Uranium is a very toxic and radioactive element. Removal of uranium from wastewaters requires remediation technologies. Actual methods are costly and ineffective when uranium concentration is very low. Little is known about the enhancement of sorption of uranyl ions by phosphate ions on aluminosilicates. Here, we studied sorption of uranyl acetate on red clay in the presence of phosphates. The concentration of U(VI) ranged 0.0001–0.001 mol/L, whereas the concentration of PO_4_^3−^ was constant at 0.0001 mol/L. We designed a new method for the analysis of ternary surface complexes. We observed for the first time a remarkable improvement of U(VI) sorption on red clay under the influence of phosphates. We also found that at least two different ternary surface complexes U(VI)–phosphate–clay are formed in the sorbent phase. The complexation of UO_2_^2+^ cations by phosphate ligands in the sorbent phase was confirmed by the X-ray photoelectron spectra of U 4f electrons.

## Introduction

Uranium is a very toxic and radioactive element. Low levels of it occur naturally in all rocks, soils, and waters (Shawky et al. 2005). Uranium present in the biosphere originates from reprocessing of uranium ores in the production of nuclear fuel and the use of depleted uranium in military applications (Bleise et al. 2003). High concentrations of uranium are found in wastewaters near processing facilities.

Removal of uranium from wastewaters requires remediation technologies. Several methods are applied to eliminate uranium from wastewaters and process effluents. The best known among them are reduction followed by chemical precipitation, ion exchange, electrochemical precipitation, solvent extraction, membrane separation, and biosorption (Konstantinou and Demetriou 2007). These methods, however, are costly and ineffective when uranium concentration is very low (Blázquez et al. 2005). Sorption of uranium on different mineral adsorbents is an effective alternative method for its removal. Until now, the results of several experimental studies have been published in this field (Aksoyoglu 1989; Ames et al. 1983; Arnold et al. 1998; Donat and Aytas 2005; Fuller et al. 2003; Giammar 2001; Hongxia and Zuyi 2002; Kilincarslan and Akyil 2005; Baumann et al. 2005; Wersin et al. 1994; Waite et al. 1994; Zhijun et al. 2004; Zuyi et al. 2000).

Little is known about the enhancement of sorption of uranyl ions by phosphate ions on aluminosilicates. The most notable study is the dissertation of Bachmaf concerning the sorption of U(VI) on the clay minerals bentonite and kaolinite (Bachmaf 2010). That author observed a clear improvement in the kinetics of U(VI) sorption on bentonite in the presence of phosphates when the system was compared to ones in which sorption occurred in the presence of sulfates and carbonates. Unfortunately, the author did not provide any spectroscopic evidence for U(VI) complexation by phosphates in the adsorbent phase. Apart from that, no tests for the desorption of U(VI) from bentonite in the presence of phosphates were carried out. The problem is very important from the practical viewpoint, since aluminosilicates are known as potential materials for the construction of geological barriers for safe storage of nuclear wastes (International Atomic Energy Agency 2004). Here, we show, for the first time, an unequivocal improvement in U(VI) sorption on the red clay when phosphates are introduced to the aqueous phase. The complexation of UO_2_^2+^ by ≡Si–OH and ≡Al–OH surface groups and by PO_4_^3−^ ions is confirmed by XPS spectroscopy.

## Materials and methods

### Characterization of the red clay

The red clay used in this work came from the Pałęgi mine located in Kielce (Poland) and was supplied by the Geol-Min company. Its mineralogical and chemical composition is as follows: illite (23–37 %), kaolinite (6–12 %), chlorite (3–5 %), quartz (30–45 %), hematite (3–6 %), SiO_2_ 64.79 %, Al_2_O_3_ 16.26 %, Fe_2_O_3_ 7.22 %, MgO 2.38 %, K_2_O 2.68 %, CaO 0.4 %, TiO_2_ 0.91 %, MnO 0.09 % (Gładysz-Płaska et al. 2012). The sodium form of the clay (Na-clay) was obtained through contacting 5 g of raw clay with 100 cm^3^ of 1 mol/dm^3^ NaCl solution (Sigma-Aldrich, 99.5 % purity).

The cation exchange capacity (CEC) of the red clay was 0.0005 mol/g, as found by exchange with 0.005 mol/dm^3^ [Co(NH_3_)_6_] Cl_3_ (Gladysz-Plaska et al. 2012).

### Equilibrium study

The adsorption isotherms of U(VI) were determined by contacting a 0.2 g sample of Na-clay with 100 cm^3^ of UO_2_(CH_3_COO)_2_∙2H_2_O + Na_2_HPO_4_∙7H_2_O solution (Lachema, n.p., Brno, p.a., Sigma-Aldrich) at concentrations of 0.0001–0.001 mol/dm^3^ and 0.0001 mol/dm^3^ for U(VI) and phosphates, respectively. The following parameters were maintained: a mechanical shaker WU-4, shaking speed 170 oscillations/min, shaking time 6 h, and temperature 22 °C. After shaking, the samples were left to stand for 12 h and were then passed through filter paper (Filtrak 390, Polish Chemical Reagents) and centrifuged at 10,000 rpm for 15 min (Med. Instruments). The initial and the equilibrium concentrations of U(VI) in the aqueous phase were determined by the Arsenazo(III) method (Marczenko and Balcerzak 1998), whereas the equilibrium concentrations of phosphate ions in the aqueous phase were measured spectrophotometrically by the phosphomolybdic method (Marczenko and Balcerzak 1998).

The concentrations of U(VI) and PO_4_^3−^ ions in the clay phase (c_s_) in mol/g were calculated from the relationship:

$$ {\text{c}}_{\text{s}} = \, \left( {{\text{c}}_{0} {-}{\text{ c}}_{\text{eq}} } \right) \cdot {\text{V}}/{\text{m}}, $$

where c_s_, c_0_, and c_eq_ denote the concentrations of U(VI) and PO_4_^3−^ ions in the clay phase, the initial solution, and the equilibrium solution, respectively. The symbols V and m relate to the volume of solution in dm^3^ and mass in g.

Desorption of uranium from U-clay was studied in the following way: 200 mg of U-clay was shaken with 0.01 mol/dm^3^ solutions of NaCl, NaNO_3_, Na_2_CO_3_, or an HNO_3_ solution of pH 3. After 6 h, the samples were filtered and centrifuged, and concentrations of uranium in the aqueous phase were measured.

Desorption percentage was calculated from the ratio of the number of U(VI) moles in the aqueous solution to the number of U(VI) moles in a sample of clay before desorption.

### X-ray photoelectron spectroscopy

The samples for XPS spectra (X-ray photoelectron spectroscopy) analysis were prepared by shaking 1.5 g of Na-clay with 100 cm^3^ of 0.002 mol/dm^3^ UO_2_(CH_3_COO)_2_ solution or with a mixture of 0.002 mol/dm^3^ UO_2_(CH_3_COO)_2_ 2H_2_O and Na_2_HPO_4_7H_2_O (concentration of both components 0.002 mol/dm^3^). After 6 h, the mixture was centrifuged, and the solid residue was dried in the air.

U 4f XPS spectra were recorded on an ESCA apparatus with a multidetection electron analyzer Scienta R4000 (produced by VG Scienta) in the fixed analyzer transmission mode. An unmonochromatized Al*K.ψ*source (1,253.6 eV) with a voltage of 12 kV and an emission current of 30 mA was employed. Powdered samples were placed on a molybdenium sample holder and submitted to a vacuum of 5 × 10^−9^ mbar. The U4*fψ*spectra were fitted, using CASA XPS software, with a Gaussian–Lorentzian peak shape after subtraction of the background with a Shirley baseline; the uranium 4*fψ*spin–orbit coupling was maintained at 10.8 eV, and the component ratio U4*f*5)2)U4*f*7)2 was constrained to 0.75.

## Results and discussion

The aim of the investigation was the registration of the uranium(VI) adsorption/desorption isotherms on the red clay in the absence and presence of phosphates in the aqueous phase supported by the observation of X-ray photoelectron spectra of the adsorption products.

### Adsorption study

One has been found from sorption kinetics investigation that for the system with phosphate ions, the equilibrium is completed within 2 h contrary to 5 h observed for the case without phosphates. Furthermore, the kinetics for the system with the lack of phosphates is complicated. There is evident increase in the sorption percentage of U(VI) within 1 h with its successive drop after 3 h and repetitive increase with plateau attainment during 5 h. This may be the result of the quick electrostatic bonds formation between uranyl ions and negatively charged alumninosilicate skeleton of clay at the beginning of the sorption process and the slow completion of the covalent bonds formation between the uranyl ions and surface sorption sites, i.e., between ≡Al–OH and ≡Si–OH groups within 5 h.

**Fig. 1:**
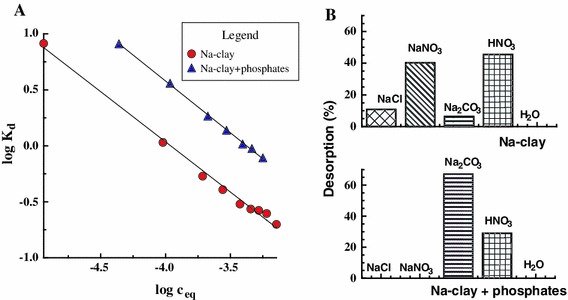
**A** Sorption isotherms of U(VI) ions (liner model equation: *filled triangle* : log K_d_ = −0.93 log c_eq_–3.13; *r* ^2^ = 0.999; *filled circle*: log K_d_ = −0.90 log c_eq_–3.55; *r* ^2^ = 0.994), and **B** U(VI) desorption from Na-clay in the absence and presence of phosphates (the values of the U(VI)/PO_4_^3−^ molar ratio *R* for the particular points of the isotherm are as follows: 1.17, 2, 3, 3.56, 3.91, 3.88, 4.05, 4.07, 4.36, 4.38; pH_eq_ 5–5.3)

Sorption isotherms of U(VI) for both systems, i.e., for the absence and presence of phosphates, are given in Fig. 1A. The solid lines refer to the linear model based on the equation:

1 $$ {\text{log K}}_{\text{d}} = {\text{ a }} + {\text{ b\,log c}}_{\text{eq}} $$

where K_d_ and c_eq_ denote, respectively, the distribution constant of U(VI) and its equilibrium concentration in the aqueous phase.

A decrease in log K_d_ values with log c_eq_ was observed, resulting from a reduction in the number of adsorption sites on the clay surface available for U(VI) ions. The linear character of the log K_d_ versus log c_eq_ dependence most probably results from the presence of adsorption sites with similar adsorption energy.

There is an evident improvement in U(VI) sorption in the presence of phosphates. This is probably a consequence of the formation of some unknown mixed surface complexes of U(VI) with PO_4_^3−^ ions and ≡Al–OH, ≡Si–OH species. The value of the U(VI)/PO_4_^3−^ ratio R in the solid phase increases from 1.17 to 4.38 with the equilibrium concentration c_eq_ of U(VI). Therefore, it is impossible that the precipitation of (UO_2_)_3_(PO_4_)_3_ 4H_2_O is exclusively responsible for the improvement in U(VI) sorption.

The leaching of U(VI) from Na-clay is manifestly more difficult in the presence of phosphates (Fig. 1 B) than in the system without phosphates. In the system with phosphates, merely 0.01 mol/dm^3^ Na_2_CO_3_ and HNO_3_ (pH = 3) solutions desorb U(VI) from the adsorbent surface owing to the formation of strong U(VI) carbonato complexes (Giammar 2001; Grenthe et al. 1992) or as a result of replacement of Na^+^ structural ions by mobile protons. Uranyl ions are strongly bound by the sorptive sites of Na-clay and cannot be transferred to the aqueous phase via the formation of weak chloride or nitrate complexes as in the case of the system without phosphates.

**Fig. 2:**
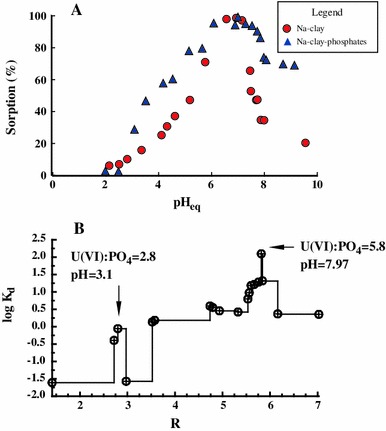
The effect of pH on U(VI) sorption (**A**) and the change in U(VI) distribution constant with the U(VI)/PO_4_ molar ratio R in the sorbent phase (**B**) (initial concentration of U(VI) 0.0005 mol/dm^3^)

The change of the sorption percentage with pH is positively modified upon the addition of phosphates (Fig. 2A). In the acidic pH range, i.e., up to pH = 7, a sharp increase in U(VI) sorption is observed, probably as a consequence of a transfer of the cationic species UO_2_^2+^ and UO_2_(H_2_PO_4_)(H_3_PO_4_)^+^ as well as the neutral species UO_2_(H_2_PO_4_)_2_ and (UO_2_)_3_(PO_4_)_3_ 4H_2_O (Guillaumont et al. 2003) from the aqueous to the sorbent phase. In the pH range of 7–9, there is only a partial decrease in the sorption percentage from 100 to 70 %, unlike in the system without phosphates, where a drop in the sorption from 100 to 20 % is observed, resulting from the presence of soluble carbonate complexes UO_2_(CO_3_)_3_^4−^ (Giammar 2001; Grenthe et al. 1992), which are repelled by the negatively charged aluminosilicate framework of the red clay.

The curve representing the change in log K_d_ with the U(VI)/PO_4_^3−^ molar ratio (Fig. 2B) has two sharp maxima at *R* = 2.8 and 5.8. This is probably a result of the presence of at least two different surface complexes of U(VI) ions with PO_4_^3−^ ions. Presumably, phosphate ligands play a bridging role in the ternary U(VI)–phosphate–clay complexes similar to that found by Singh, who studied U(VI) sorption on goethite (Singh et al. 2012). From the practical viewpoint, it is important that the sorption of U(VI) from aqueous solutions should be effective in the pH range of 7–9 in the presence of phosphates.

### X-ray photoelectron spectroscopy studies

**Fig. 3:**
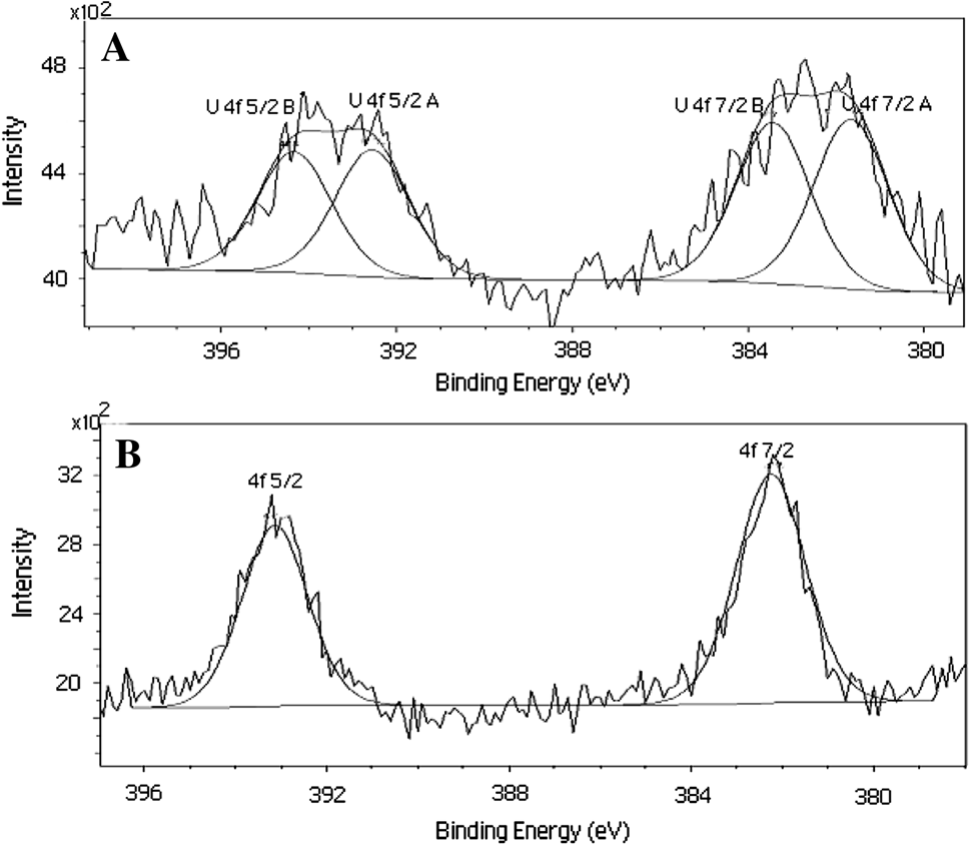
X-ray photoelectron spectra of U(VI) adsorbed on Na-clay in the absence (**A**) and presence of phosphates (**B**)

U 4f XPS spectra of the red clay samples in the absence and presence of phosphates are shown in Fig. 3. The respective peaks were decomposed into two components for the case without phosphates. The core binding energies are 381.6 and 383.4 eV for the U 4f7/2 band. The first peak probably corresponds to the binding of UO_2_^2+^ cations by aluminol sorptive sites, i.e., by ≡Al–OH groups, whereas the second one relates to the complexation of uranyl ions by silica sorptive sites, i.e., by ≡Si–OH groups. The values of binding energies are very similar to those found by Kowal for a U(VI)–montmorillonite system (Kowal-Fouchard et al. 2004). For the samples with phosphates, only one peak in the XPS spectrum is observed. The core binding energy 382.2 eV is close to 382.3 eV, which is characteristic of uranyl on a uranium oxyphosphate reference (Drot et al. 1998), which corresponds to the PO_4_^3−^ environment.

## Conclusion

The remarkable enhancement of U(VI) sorption on the red clay by phosphates opens a broad field in the investigations concerning the application of hybrids of phosphates and aluminosilicates as new materials for the construction of geological barriers. The study of surface complexation of UO_2_^2+^ ions by PO_4_^3−^, silanols, and aluminols would certainly facilitate the choice of the optimal conditions for the binding of uranyl ions by the sorbent surface.
